# Determinants of mental health professionals’ attitudes towards recovery: A review

**DOI:** 10.36834/cmej.61273

**Published:** 2020-09-23

**Authors:** Mimosa Luigi, Filippo Rapisarda, Marc Corbière, Luigi De Benedictis, Anne-Marie Bouchard, Amélie Felx, Massimo Miglioretti, Amal Abdel-Baki, Alain Lesage

**Affiliations:** 1 Department of Psychiatry and Addictology, University of Montreal, Quebec, Canada; 2 Department of Psychology, Università degli Studi di Milano-Bicocca, Italy; 3 Department of Education and Pedagogy – Faculty of the Sciences of Education, University of Quebec, Quebec, Canada; 4 Program for psychotic disorders, Institut Universitaire en Santé Mentale de Montréal, CIUSSS East-of-Montreal, Quebec, Canada; 5 Mental health and substance abuse program, Institut Universitaire en Santé Mentale Douglas, CIUSSS West-of-Montreal, Quebec, Canada; 6 Research Centre, Institut Universitaire en Santé Mentale de Montréal, CIUSSS East-of-Montreal, Quebec, Canada

## Abstract

**Objective:**

The attitudes of mental health professionals towards consumers’ recovery are far more pessimistic than what is needed for the recovery-orientation to truly permeate systems of care. It has become pressing to depict determinants for these attitudes and how they evolve during professionalization. This, in the hopes to adjust not only medical education, but also ongoing training of professionals.

**Methods:**

A systematic search of PubMed and PsycINFO databases was conducted, yielding a net 15 303 records. Twenty-two publications from specific educational journals and reference lists were added. Finally, thirty-four full texts were read, from which twenty-two articles were included.

**Results:**

From the reviewed studies emerged five main determinants: profession, education, age, clinical experience, and nature of the contact with consumers. Traditional clinical placements during residency, negative experiences with acute patients, younger age and the professional attitudes of psychiatrists seem to all be determining factors for professionals’ pessimistic attitudes towards recovery.

**Conclusions:**

This review found specific determinants for attitudes in recovery and four out of five can be acted upon. For a recovery-orientation to be implemented across our mental health system, we formulate recommendations within the Canadian context for revision of curriculum, recovery-specific training, and operationalisation through state/provincial technical assistance centers.

## Abstract (fr)

**Objectif:**

L’attitude des professionnels en santé mentale envers le rétablissement des clients est beaucoup plus pessimiste qu’il ne le faut pour qu’une approche axée sur le rétablissement s’intègre réellement aux systèmes de soins. Il est urgent de décrire les facteurs déterminants de ces attitudes et la façon dont ils se développent durant le parcours professionnel, dans l'espoir d’adapter non seulement l’enseignement de la médecine, mais également la formation continue des professionnels.

**Méthodes:**

Une recherche systématique des bases de données PubMed et PsycINFO a été réalisée et a identifié 15 303 enregistrements nets. Vingt-deux autres publications ont été incluses, provenant de revues spécifiques en éducation médicale et des listes de référence. La lecture des titres et résumés des enregistrements a retenu 34 articles. Après lecture intégrale de ces derniers, vingt-deux articles ont été sélectionnés pour cette revue.

**Résultats:**

L'examen des études a révélé cinq principaux facteurs déterminants : la profession, l’éducation, l’âge, l’expérience clinique et la nature du contact avec les clients. Les stages cliniques traditionnels durant la résidence, les mauvaises expériences avec les patients en phase aiguë, un âge plus jeune et les attitudes professionnelles des psychiatres semblent tous être des facteurs déterminants de l'attitude pessimiste qu'entretiennent les professionnels envers le rétablissement.

**Conclusions:**

Cette revue de littérature a révélé des facteurs déterminants précis en ce qui a trait aux attitudes envers le rétablissement et il est possible de donner suite à quatre sur cinq d'entre eux. Afin de mettre en œuvre une approche axée sur le rétablissement dans l’ensemble de notre système de soins en santé mentale, nous avons formulé des recommandations pour la révision des programmes d’enseignement applicables au contexte canadien, la formation centrée sur le rétablissement et l’opérationnalisation de ces recommandations par l'entremise de centres de soutien technique dans les états ou provinces.

## Introduction

Professionals’ attitudes have an important impact on both the process and outcome of mental health (MH) care. MH practitioners bring together knowledge and representations of mental illness from policy makers, academics, lay people, and the media.^1^ Nevertheless, they are likely to hold the same stigmatizing attitudes towards those affected by mental illness as the general population.^2^^,^^3^ In fact, consumers themselves have identified that contact with MH services can be one of the most stigmatizing experiences of their illness^4,^^5^ and one in four users will experience such stigma.^5^ Stigmatizing attitudes in professionals, i.e. “iatrogenic stigma”, is thought to be comprised of both low expectations for prognosis and for a consumer’s character and attributes.^6^ Such negative biases can cause professionals to encourage low-risk activities and compliance, thus removing responsibility from consumers and reducing empowerment;^5^ both of which further delay their recovery process.^6^^,^^7^ Moreover, there is a tendency for providers to hold low expectations of consumers’ motivations.^8^ For example, staff repeatedly state consumer-related aspects (i.e. lack of motivation or insufficient cognitive levels, symptoms, etc.) as the main barriers to successful recovery measures.^2^^,^^4^^,^^9^^,^^10^ Finally, at an organizational level, leading staff’s attitudes will influence the recovery orientations of programs^11^ and the degree of implementation of new measures.^12^ In all these ways, professionals’ attitudes towards service consumers can have a greatly detrimental effect on their recovery journey.

This concept of recovery in MH care has been debated through the last decades and its definition could vary among different professional and non professional social groups. Still, because of the deinstitutionalization of psychiatric care in the 1990s, the physical disability and addiction movements, and most importantly the activism of “consumer-survivors”, the concept of *personal* recovery has emerged as opposed to the *biomedical* model of recovery;^13^^,^^14^^,^^7^^,^^15^ the latter being more of an approximation of “cure”, with clinical outcomes (i.e. reduction of symptoms) mostly set up by professionals rather than consumers themselves.^13^^,^^4^^,^^7^^,^^15^^,^^16^ Although no present consensus exists in defining personal recovery, a recent systematic review has identified four re-occurring components: that it is an *individualized/person-centered* process, and that it’s anchored in constructs of *empowerment, purpose*, and *hope*.^17^ Through these and other constructs, a consumer may reclaim one’s identity but also “recover a life”, regaining the right to participate in economic and civil facets of their community.^16^

Although the recovery movement has gained a lot of attention in the past decade, there are still numerous barriers to implementing recovery practices, including professional attitudes.^18^^,^^19^^,^^11^ Canada has attributed growing importance to recovery in reports such as the Mental Health Commission of Canada’s (MHCC) *Changing directions, changing lives: The mental health strategy for Canada*.^20^ But this document presents few recommendations for changing attitudes, rather simply reiterating the importance of ongoing training and pointing at contact-based education to break down stigma.^20^ However, based on studies regarding the decline of empathy and patient-centeredness through medical residency,^21^^-^^23^ it’s probable that attitudes towards recovery may already worsen in medical students throughout their education as they come in contact with more service users. These findings pose the questions: when and how should we train our MH professionals in personal recovery?

The need for training our providers differently and evaluating programs based on best-evidence to overcome this “implementation deficit disorder” has been echoed in following assemblies of experts such as the 2014 *Consensus Statement On Improving Mental Health Transitions*.^24^ This consensus had endorsed the development of Provincial Technical Assistance Centers (PTAC), inspired by the US “technical assistance centers”: state/provincial bodies entrusted to approve the creation and maintenance of community care teams, support their implementation and training, monitor quality, and evaluate results.^25^ An example of such an organisational strategy is the National Centre of Excellence in Mental Health in Quebec. Through its partnership with the Center for Studies on Rehabilitation, Recovery and Social Inclusion (CÉRISS), it offers training, consultations, and coaching on best practices in recovery.

In order to better inform these training programs, there remains a need to understand better how professionals’ attitudes toward recovery develop, in which phase of the education and professionalization process they emerge, and which factors mediate their development. Our literature review is thus intended to assist these implementation efforts in recovery work by depicting specific determinants for professionals’ attitudes towards recovery.

## Methods

M.L conducted a review of literature in May and July 2017 using PubMed and PsycINFO databases. Figure 1 presents a diagram of our methodology. The multiple search terms chosen were grouped into four categories, each representing a factor from the research question: mental health, recovery, staff, and professional culture (see Supplementary Table 1). Note that although this paper presents the results pertaining to staff attitudes, a larger array of terms thought to define professional culture were originally used. The conjunction “AND” served to link groups while ‘’OR’’ separated related terms. Truncation was used when possible. The limits added to the search were French and English languages, ‘Journal articles’(PubMed) or ‘Peer-reviewed journal’(PsycINFO), ‘Literature review’(PsycINFO) or ‘Systemic reviews’(PubMed), and publication dates since 1980. Finally, the research for peer reviewed journals in PsycINFO included an added ‘Psychosocial Rehabilitation’ index term.

Out of the 15 303 results, articles were included for abstract reading if the title made mention of attitudes or one aspect of professional culture. Most articles were excluded at this step because of study populations other than MH professionals (solely consumers or general population, caretakers, general medical staff, etc.). Other exclusion criterion for this review were any physical or mental illness other than an MH diagnosis, studies regarding the evaluation of instruments and practices, recommendations for recovery interventions and practice, medication trials, cognitive rehabilitation therapies, studies on peer support, consumer narratives, post-disaster studies, spirituality in recovery or self-stigma. Qualitative studies were also excluded in order to focus this review on statistically significant determinants of the recovery/professional relationship. Thirty-four articles were retained at this step. Finally, a hand search through reference lists and related publications yielded 19 more articles for full text reading.

Further, as some of the literature seemed to make the distinction between the attitudes of professionals and those of trainees/residents, a second PubMed search was conducted using the word “recovery” in titles or abstracts of publications from seven journals with a focus on medical education. These journals were chosen for their impact factors in psychiatry (see Figure 1). This served to verify that key articles had not been omitted from the first research and assess what has already been written concerning recovery and rehabilitation education in psychiatry. Only three relating articles met the aforementioned inclusion criteria for attitudes, this time in residents and trainees. This made for a total of 56 articles selected for full-text reading: 34 from the original search, 19 from reference lists, and three from educational journals.

M.L independently screened and read all articles. Co-authors did not put forth any missing articles apart from works further defining precise psychiatric terms. M.L carried out the extraction of information after full-text reading in standardised extraction tables. All co-authors contributed to the analysis as well as the step by step validation of the paper. Articles excluded at this step did not study recovery attitudes through any scale or questionnaire item despite having mentioned general attitudes towards mental illness in their abstracts. The final number of publications retained for this review was 22: 18 studies and four reviews.

Only five^15^^,^^26^^-^^29^ out of 18 studies retained for the results defined recovery either in their introductions or through their scale and questionnaire items. These five studies emphasized the individuality of recovery as a process, generally adhering to Antony’s personal recovery definition or a variant of it: *A journey of self-discovery, a unique personal process of changing one’s attitudes, values, feelings, and goals, and involves finding new meaning of life with or without the limitations caused by mental illness.”*^30^

**Papers’ selection process**

**Figure:**
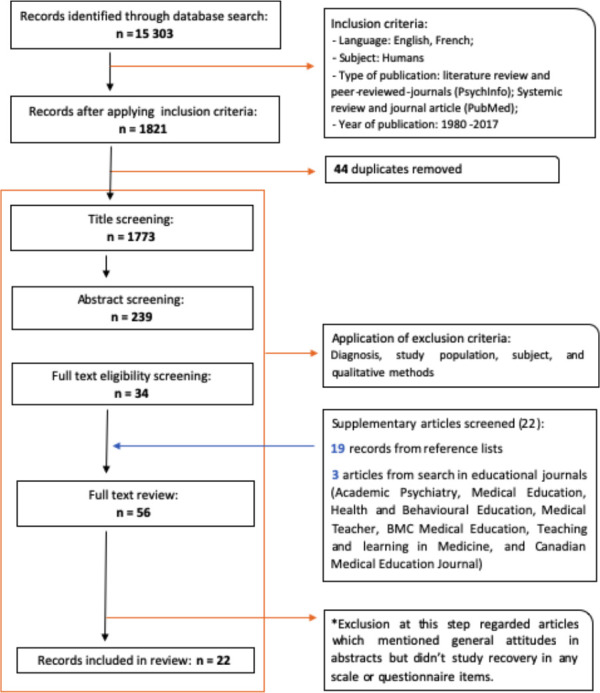


## Results

As research regarding MH professionals’ views of recovery is limited, most of our findings are derived from studies on general attitudes regarding MH in which service providers were part of or were the main population examined. Attitudes towards recovery specifically were partially investigated in questionnaires as well as beliefs and values scales from the selected studies. From these findings emerged five main determinants for attitudes towards recovery.

### Type of profession

Some studies did not find significant correlations between type of profession and attitudes towards MH in general.^31^^,^^32^ Moreover, our review did not uncover any research specifically aimed at comparing attitudes in different professionals and many studies did not have samples sufficiently representative of different disciplines to allow for such comparison. Still, the available research suggests that psychiatrists and physicians in general have more negative attitudes about psychiatric rehabilitation principles (PRP) than other professionals. Inversely, nurses seem to hold the most positive attitudes even when the overall sample of professionals is pessimistic about PRP. Only one study^33^ contradicted this tendency: Italian psychiatrists holding more positive views about schizophrenic patients’ social functioning than psychiatric nurses.^4^ Nevertheless, in a study on 743 Taiwanese professionals from different MH service settings, doctors agreed and enacted less with PRP than occupational therapists.^15^ Type of profession was incidentally found to be a significant predictor of PRP enactment. Two more studies^34^^,^^35^ found the same trend for outcomes in a clinical vignette describing schizophrenia^3^ and the Caldwell and Jorm study, for views about outcomes for both schizophrenia and depression.^4^ Still, such rankings do not mean that MH nurses only hold positive attitudes. Ross and Goldner’s literature review on nurses’ attitudes makes the point that although psychiatric nurses hold more optimistic views of prognosis and outcomes than the public and other professionals, the negative attitudes of some could be linked to their discontent for the medical model and support for alternative treatments.^36^ Although not as well documented, there also seems to be a trend of clinical psychologists being the group with the most favourable views of psychiatric recovery after psychiatric nurses; psychologists composing the second less stigmatizing group in the above-mentioned Caldwell and Jorm study.^34^ Moreover, in a large sample of 1073 MH professionals in Switzerland, psychiatrists held the most stigmatizing views regarding MH and psychologists, the most positive.^37^ Finally, although the Peris et al.’s study did not find a statistically significant correlation between attitudes and type of profession, clinical psychologists were still found to hold more positive attitudes towards individuals with mental illness and their recovery outcomes.^26^ Thus, professional discipline seems to be an important determinant of professional attitudes towards recovery.

### Age, education, and experience

Still, these attitudes are a result of more than professional affiliation; they are thought through the course of one’s higher education in the field and are influenced by peers and superiors, the literature read, the conferences one has attended, and a clinician’s experiences in specific settings. All studies reviewed except one^37^ found that age, experience, and education had some level of effect on attitudes towards MH and recovery. The extent to which these factors have an influence is unclear; which is why this review attempts to summarize the available literature on the subject.

Regarding *age*, research on ACT teams in the US found that older age significantly and positively impacted implicit biases about MH.^38^ In Bjorkman et al.’s study, increased age in psychiatric nurses from a Swedish hospital had a significant and positive effect on attitudes about the prospect of recovery from severe depression and panic attacks.^49^ Casper and al. also suggested that age might be a predictor for attitudes in psychiatric rehabilitation.^15^ Finally, Song’s study significantly linked age as a predictor for the enactment of PRP (*ibid*.)

### Education and training

Education and training within specific disciplines or settings might also predict attitudes towards recovery. In two studies, psychiatric nurses were found to have more positive attitudes towards recovery and MH if they had advanced diplomas or training.^27^^,^^40^ Song’s review concluded that attending recovery-related courses was significantly predictive of the enactment of PRP.^15^ In Casper and al.’s work, degree and literature read were both predictors of attitudes towards recovery.^15^^,^^31^ Concerning their measure of literature read, professionals in this study were asked how many psychiatric rehabilitation authors they had read from the following list: Anthony, Carling, Liberman, Bond, Dincin, Rutman, Deegan, and Solomon.^31^ In addition, Casper and Oursler’s 2003 evaluation of the Psychiatric Rehabilitation Beliefs, Goals and Principles Scale linked academic degrees to positive initial scores.^28^ Moreover, a study conducted in two state hospitals from Indiana examined changes in attitudes about recovery resulting from two types of recovery training: general/inspirational and specific/practical training.^41^ This research measured professionals’ expectations for consumers, for their own professional capacity, and to what degree they implemented recovery practices. At the one year follow up, professionals with higher levels of education had higher consumer optimism scores but lower scores on factors such as involving consumers in staff trainings, clients’ choice regarding their own care, and the degree to which services were tailored to consumers. Specific/practical training created longer lasting effects, a greater increase in providers’ efforts to help clients pursue their life goals, and a greater improvement of staff’s beliefs in their hospital’s recovery-orientation. Lastly, only in Stull et al.’s study were higher levels of education negatively and significantly related to implicit bias about mental illness in general.^38^ The following section calls into question whether this last finding is an outcome of experiences with consumers during professionalization rather than the content of the education received.

### Clinical experience

Despite Lauber et al.’s finding that experience has little effect on stereotypes about MH^37^, clinical experience will invariably influence attitudes held by professionals towards a clientele they know. What is still undetermined about experience is if it increases or decreases positive attitudes. This is partly due to the fact that when comparing medical students at different stages of their residency, education and clinical experience become intertwined in their impact. One Italian study found no significant difference between 1st and 5th/6th year students in their belief that patients with schizophrenia “would be well again.”^42^ Nevertheless, more research seems to suggest that the clinical placements/psychiatric rotations undergone during medical residencies have adverse effects on beliefs about recovery. A study in Pakistan found that doctors hold more optimistic views of recovery than students that have undergone recent clinical placement.^43^ These students’ attitudes were also more pessimistic than students’ in their pre-clinical years. This difference between 1st and 6^th^ year was also uncovered in 100 medical students from Japan when asked if schizophrenic patients could recover if treated at an early stage.^29^ Moreover, in evaluating the attitudes of 12 trainee psychiatrists in Hong Kong, it was also observed that paternalistic and pessimistic views of recovery became more risk-averse and biomedically oriented with their clinical experience.^8^ One Swedish study interestingly paralleled this finding in nursing staff: recently trained staff had more negative attitudes about the prospect of recovery with regards to severe depression.^39^ From all this literature seems to emerge the notion that recent clinical placements may have negative effects on attitudes about recovery in trainees.

It remains unclear what effect experience as a professional has. Some studies indicate a positive effect:^28^^,^^39^^,^^44^ either by comparing students to professionals^44^ or measuring a significant effect of experience on attitudes.^28^^,^^39^ Inversely, Tsai et al.’s study found that staff with more years in their position have significantly lower expectations for their clients.^41^ Another study reported that clinical psychologists have more negative attitudes towards MH than graduate psychology students.^32^

### Nature of contact

According to the contact hypothesis, more exposure and contact with persons presenting a mental illness should increase positive attitudes towards these clients. This theory could explain why staff in direct or repeated contact with consumers have been found to hold more positive explicit attitudes towards them than managers.^38^ It also gives sense to the finding that MH staff caring for schizophrenic patients said to be “recovered” will hold more psychosocial views of recovery interventions;^8^ views which are in line with outcomes most important to patients themselves. An alternative explanation is that those with more favorable attitudes choose to work closely with patients and to take part in more rehabilitation efforts.^38^ In any case, as the findings concerning experience show, contact with acutely ill patients may have the opposite effect; thus making the contact hypothesis valid only for certain types of interactions with consumers. In reality, clinical experience in hospitals seems to reinforce the beliefs of chronicity and incurability of mental illnesses such as schizophrenia.^45^^,^^41^ Such negative contact also explains how medical students’ psychiatric rotations working with acutely ill in-patients would create or exacerbate negatives views of recovery. In their review on attitudes about MH in the nursing profession, Ross and Goldner reported that most studies attributed pessimistic expectations for prognoses and outcomes to contact with the most dysfunctional and chronically ill of consumers.^36^ Thus, it would not only be about experience but the nature of the contact with consumers. Just as iatrogenic stigma leads to what Thornicroft termed “physician bias” (overly pessimistic views as a result of an acutely ill caseload), negative clinical experiences would lead to Harding and Zahnister’s “clinician’s illusion”: seeing the most clinically ill as typical caseloads when in fact, they are not the norm.^4^^,^^6^^,^^46^

## Discussion

This review has presented an up to date appraisal of the available literature on determinants of MH staff’s attitudes towards recovery. Overall, the results conclude that MH professionals hold negative views about the prospect for recovery, either in biomedical or personal recovery terms as articles differed in their definition. This would in part be due to what we call the nature of contact during training rotations. Professionals entertain these beliefs despite longitudinal studies showing that over 50% of persons diagnosed with schizophrenia or psychosis can achieve favourable outcomes.^6^^,^^7^^,^^14^^,^^45^^,^^46^ Additionally, psychiatrists seem to present the most pessimistic views. On the other hand, nurses, followed by psychologists, exhibit the most optimistic. A likely explanation lies in the guiding orientation of these professional groups’ training, with nurses possibly placing a greater emphasis on holistic care and relying less on the medical model than other professional groups.^34^^,^^36^ Moreover, much more frequent contact with patients could enable nurses to witness more subtle improvements. Long term care nursing staff could therefore remain hopeful despite managerial scepticism, and would benefit from better recognition and empowerment.^47^ That providers can hold overall positive attitudes about consumers does not protect from pessimism about recovery potential.^40^^,^^4^^8^ Finally, age and specific recovery training would both positively impact attitudes whilst negative clinical experience could work against these factors.

## Limitations

The first limitation of this review is that reading and quality assessment were only carried out by one reviewer (M.L). However, all co-authors are considered experts in the fields of recovery and rehabilitation both in clinical and educational settings (through specific work on recovery and teaching related practices to residents). Secondly, the search only included two scientific databases. Still, an important volume of publications (15 303) were screened and none of the co-authors identified additional articles. Findings from across America, Asia, Australia, and Europe were reviewed giving our results a good generalizability. The studies compiled were also conducted in community settings, hospitals, and universities. As systems of care and beliefs regarding MH are greatly influenced by culture and organizational factors, such diversity represents an important strength of this review. Finally, selected studies did not encompass all MH diagnoses as literature has mostly focused on psychotic disorders. These may be the main diagnoses related to negative attitudes as severity of symptoms seems to be proportional to the degree of negative attitudes.

Our review has reconfirmed that both attitudes in professionals and the gap between principles and enactment in recovery work should be addressed through continuous investment into what Thornicroft and Tansella termed “human technology”; which MH care relies so heavily on.^48^ What’s encouraging is that programs specifically geared towards recovery knowledge can positively impact all professionals. In Casper and Oursler’s measures of recovery knowledge before and after PRP training, knowledge gains were equivalent for staff with different degrees.^28^ Moreover, significantly higher scores on their psychiatric rehabilitation scale were resulting of the curriculum and not individual characteristics. These findings tell us that recovery requires a curriculum of its own, from education to ongoing training. In fact, Thornicroft and Tansella had cautioned, in the *Mental Health Matrix*, that experience does not necessarily produce expertise.^4^^9^ This is, in all evidence, especially true for rehabilitation work.

### Implications for education

Gordon et al. reported that 6^th^ year medical students, during their first tutorial on recovery, estimated that only 0 to 20% of consumers would recover.^50^ Even more unsettling: when given the research stating that it was rather half to three quarters of schizophrenic patients who can recover, students said that this was contradictory to their previous instruction. It follows that curriculum needs to be adjusted through more evidence-base recovery knowledge if medical students are to hold realistic expectations of recovery.

In Canada, important advancements were made in this regard. In 2009, the Canadian Psychiatric Association published a new psychiatric residency curriculum, which was associated with a revision by the Royal College of Physicians and Surgeons in 2011.^5^^1^^,^^5^^2^ This was the first time the Royal College acknowledged education in psychiatric rehabilitation and included it in its curriculum. Nevertheless, it too has been criticized for not allocating enough time to recovery training and not including graduate level texts on psychiatric rehabilitation.^5^^1^ Attention needs be given as to how to modify curriculum since research has shown workshops, panel discussions, and focus groups can improve recovery knowledge, but only moderately affect optimism.^5^^3^ Moreover, a randomized trial has shown that a one-time contact-based educational intervention is, on its own, ineffective in changing attitudes in medical students.^5^^4^

Furthermore, it has been said that contact with service users is more effective than education or activism in reducing stigma in adults; our findings do not seem to support this claim. Trainee psychiatrists’ and medical students’ traditional rotations contribute to developing sceptical or negative expectations for recovery and towards the expert status of service users.^10^^,^^39^^,^^5^^3^ This seems to be due to the chronicity and acuteness of the mental illnesses presented by the in-patients with which residents and students often have their first and only contacts during first-year training in hospitals. Research does in fact show that attitudes in hospital settings are more negative and resistant to change than in community care.^41^ Hence the need for clinical placements in different settings to allow trainees to observe varying degrees of mental illness and form more representative beliefs regarding recovery. These rotations should also be longer as it requires time to accompany a consumer till the point where significant improvements in his recovery process are observable. A case control study in Connecticut found that a 3-month psychiatric rotation had no effect on optimism.^5^^5^ As an example of longer rotations, the University of Montreal’s general psychiatry internship for second-year psychiatry residents lasts at least a year and must be completed mostly in community services for general psychiatry (as opposed to ultra-specialised teams). It must include regular supervision, frequent follow-up visits and the elaboration of a treatment plan with multiple interventions from supported housing to psychotherapy and medication. This is supplemented by a full course on psychiatric rehabilitation in the same year and the supervision of such an internship during 3^rd^ year in a series of grand rounds presented by residents, with academic psychiatrists and peer-support workers as experts. Finally, a three to six-month internship in recovery with patients suffering from SMI is also required.

### Implications for current professionals

Ongoing training which is specific to recovery needs to be implemented for all professionals if attitudes in recovery are to change and allow for a real re-orientation of practices in multidisciplinary teams. Recovery-specific workforce training at all levels has constituted a major priority for commonwealth countries in their such reforms.^14^ The Ministry of Health and Social Services of Quebec, amongst other initiatives for adopting the National Strategy, has entrusted the role of training professionals in recovery to the Quebec Association for Psychosocial Rehabilitation (AQRP). The AQRP also trains administrators and offers follow ups and coaching. Indeed, the need for leadership’s support in applying recovery measures and standardising practices has been identified within Canadian teams.^12^^,^^5^^6^ The Ministry has also set up, in collaboration with academic researchers, a Technical Assistance Center^5^^7^ for continuous training of MH professionals across the province. Known as the CÉRISS, it offers webinars concentrating recovery education for intensive case management teams. There is preliminary evidence for the effectiveness of such self-learning programs adapted to practice settings.^5^^8^ One major advantage being the flexible delivery of a self-taught program utilizing manuals and interactive videos.

As with all the practices, rotations, and curriculum adjustments proposed, it will also be crucial for there to be ongoing formal evaluations of programs to assure care teams do not simply revert to what they know.^5^^9^ Myra Piat and other Canadian recovery experts have undertaken a mixed studies systematic review on the operationalisation of recovery into MH services for adults with SMI.^60^ The MHCC’s *Opening Minds* project^6^^1^ has also begun to evaluate a few existing training programs in Canada. Finally, although validation and consideration of scale items are still needed, numerous instruments to evaluate attitudinal change and service orientation towards recovery already exist,^6^^2^^,^^6^^3^ including those developed by the MHCC.^6^^4^ Technical centers could help disseminate these measures for evaluation of both educational and ongoing training programs.

### Conclusion

Research shows that professionals can hold negative attitudes, and many doubt the possibility for recovery. Psychiatrists represent the most pessimistic of professional groups while psychiatric nurses are the most optimistic. Age, education, and experience also have non-negligible effects on these attitudes; all factors which could benefit from closer examination. As exemplified in other commonwealth countries, a reform of MH care systems will necessitate more emphasis on recovery in residency curriculums (i.e. graduate level texts and specific courses) and ongoing training of staff at all levels on recovery prospects, principles, and evidence-based practices within their work context. Psychiatric residents also need to be given the opportunity to witness positive recovery experiences through rotations that are longer and more diverse. Lastly, both implementation and evaluation of such continuing training programs can be carried out through provincial/state technical assistance centers.

## References

[1] Morant N Social representations and professional knowledge: the representation of mental illness among mental health practitioners. The British Journal of Social Psychology. 2006;45(Pt 4):817-38. 10.1348/014466605X81036 17393882

[2] Chester P, Ehrlich C, Warburton L, Baker D, Kendall E, Crompton D What is the work of recovery-oriented practice? A systematic literature review” *International Journal of Mental Health Nursing*. 2016; 25(4):270-85 10.1111/inm.12241 27381002

[3] NSW Consumer Advisory Group - Mental Health Inc. (NSW CAG), MHCC. Literature review on recovery: developing a recovery oriented service provider resource for community mental health organisations. 2009

[4] Schulze B Stigma and mental health professionals: a review of the evidence on an intricate relationship. Abingdon, England: International Review of Psychiatry. 2007;19(2):137-55 10.1080/09540260701278929 17464792

[5] Bates L, Stickley T Confronting Goffman: how can mental health nurses effectively challenge stigma? A critical review of the literature. Journal of Psychiatric and Mental Health Nursing. 2013;20(7):569-75. 10.1111/j.1365-2850.2012.01957.x 22906050

[6] Berry C, Gerry L, Hayward M, Chandler R Expectations and illusions: a position paper on the relationship between mental health practitioners and social exclusion. Journal of Psychiatric and Mental Health Nursing. 2010;17(5):411-21. 10.1111/j.1365-2850.2009.01538.x 20584238

[7] Roberts G, Wolfson P The rediscovery of recovery: open to all. Advances in Psychiatric Treatment. 2004;10(1):37-48. 10.1192/apt.10.1.37

[8] Morera T, Pratt D, Bucci S Staff views about psychosocial aspects of recovery in psychosis: A systematic review. Psychology and Psychotherapy: Theory, Research and Practice. 2016;90(1):1-24. 10.1111/papt.12092 27239949

[9] Chinman MJ, Allende M, Weingarten R, Steiner J, Tworkowski S, Davidson L On the road to collaborative treatment planning: Consumer and provider perspectives. The Journal of Behavioral Health Services & Research. 1999;26(2):211-8. 10.1007/BF02287492 10230148

[10] Salyers MP, Rollins AL, McGuire AB, Gearhart T Barriers and facilitators in implementing illness management and recovery for consumers with severe mental illness: trainee perspectives. Administration and Policy in Mental Health. 2009;36(2):102-11. 10.1007/s10488-008-0200-0 19096924

[11] Le Boutillier C, Slade M, Lawrence V, et al Competing Priorities: Staff Perspectives on Supporting Recovery. Administration and Policy in Mental Health and Mental Health Services Research. 2015;42(4):429-38. 10.1007/s10488-014-0585-x 25134949

[12] Piat M, Lal S Service providers' experiences and perspectives on recovery-oriented mental health system reform. Psychiatric Rehabilitation Journal. 2012;35(4):289-96. 10.2975/35.4.2012.289.296 22491368 PMC4829387

[13] Carpenter J Mental health recovery paradigm: implications for social work. Health & Social Work. 2002;27(2):86-94 10.1093/hsw/27.2.86 12079172

[14] Piat M, Sabetti J The development of a recovery-oriented mental health system in Canada: What the experience of commonwealth countries tells us. Canadian Journal of Community Mental Health. 2009;28(2):17-33. 10.7870/cjcmh-2009-0020 27099409 PMC4835232

[15] Song LY The attitudes towards and enactment of psychosocial rehabilitation principles: discrepancies and correlates. The International Journal of Social Psychiatry. 2007;53(3):232-46 10.1177/0020764006074558 17569408

[16] Slade M, Amering M, Farkas M, et al Uses and abuses of recovery: implementing recovery-oriented practices in mental health systems. World Psychiatry. 2014 2 13(1):12-20. 10.1002/wps.20084 24497237 PMC3918008

[17] Ellison ML, Belanger LK, Niles BL, Evans LC, Bauer MS explication and definition of mental health recovery: a systematic review. Administration and Policy in Mental Health. 2018;45(1):91-102. 10.1007/s10488-016-0767-9 27709376

[18] Vandewalle J, Debyser B, Beeckman D, Vandecasteele T, Van Hecke A, Verhaeghe S Peer workers' perceptions and experiences of barriers to implementation of peer worker roles in mental health services: A literature review. International Journal of Nursing Studies 2016;60:234-50. 10.1016/j.ijnurstu.2016.04.018 27297384

[19] Whitley R, Gingerich S, Lutz WJ, Mueser KT Implementing the illness management and recovery program in community mental health settings: facilitators and barriers. Psychiatric Services. 2009;60(2): 202-209 10.1176/ps.2009.60.2.202 19176414

[20] Mental Health Commission of Canada. Changing directions, changing lives: the mental health strategy for Canada. 2012

[21] Hojat M, Vergare MJ, Maxwell K, et al The devil is in the third year: a longitudinal study of erosion of empathy in medical school. Academic Medicine: Journal of the Association of American Medical Colleges. 2009;84(9):1182-91 10.1097/ACM.0b013e3181b17e55 19707055

[22] Bombeke K, Symons L, Debaene L, De Winter B, Schol S, Van Royen P Help, I'm losing patient-centredness! Experiences of medical students and their teachers. Medical Education. 2010;44(7):662-73 10.1111/j.1365-2923.2010.03627.x 20636585

[23] Triffaux JM, Tisseron S, Nasello JA Decline of empathy among medical students: Dehumanization or useful coping process? Encephale. 2019 2 45(1):3-8. 10.1016/j.encep.2018.05.003 29960682

[24] Lesage AD, Bland R Consensus statement on improving mental health transitions. IHE Report. Edmonton (Alberta): Institute of Health Economics, 2014 11 Report No.: ISBN Print: 978-1-926929-24-8, Online: 978-1-926929-25-5, http://ihe.ca/publications/imht_cdc_consensus_statement_en [Accessed August 6, 2017]

[25] Briand C, Menear M Implementing a continuum of evidence-based psychosocial interventions for people with severe mental illness: part 2-review of critical implementation issues. Canadian Journal of Psychiatry. 2014 4 59(4):187-95. 10.1177/070674371405900403 25007111 PMC4079132

[26] Wahl O, Aroesty-Cohen E Attitudes of mental health professionals about mental illness: a review of the recent literature. Journal of Community Psychology. 2010;38(1):49-62 10.1002/jcop.20351

[27] Tay SE, Pariyasami S, Ravindran K, Ali MI, Rowsudeen MT Nurses' attitudes toward people with mental illnesses in a psychiatric hospital in Singapore. Journal of Psychosocial Nursing and Mental Health Services. 2004;42(10):40-7 10.3928/02793695-20041001-08 15543671

[28] Casper ES, Oursler JD The Psychiatric Rehabilitation Beliefs, Goals, and Practices Scale: Sensitivity to change. Psychiatric Rehabilitation Journal. 2003;26(3):311-4 10.2975/26.2003.311.314 12653453

[29] Mino Y, Yasuda N, Kanazawa S, Inoue S Effects of medical education on attitudes towards mental illness among medical students: a five-year follow-up study. Acta Medica Okayama. 2000;54(3):127-32. 10925737 10.18926/AMO/32304

[30] Anthony WA Recovery from mental illness: The guiding vision of the mental health service system in the 1990s. Psychosocial Rehabilitation Journal.1993;16(4): 11-23 10.1037/h0095655

[31] Casper ES, Oursler J, Schmidt LT, Gill KJ Measuring practitioners' beliefs, goals, and practices in psychiatric rehabilitation. Psychiatric Rehabilitation Journal. 2002;25(3):223-34 10.1037/h0095020 11859995

[32] Peris TS, Teachman BA, Nosek BA Implicit and explicit stigma of mental illness: links to clinical care. The Journal of Nervous and Mental Disease. 2008;196(10):752-60 10.1097/NMD.0b013e3181879dfd 18852619

[33] Magliano L, Fiorillo A, De Rosa C, Malangone C, Maj M Beliefs about schizophrenia in Italy: a comparative nationwide survey of the general public, mental health professionals, and patients' relatives. Canadian Journal of Psychiatry. 2004;49(5):322-30. 10.1177/070674370404900508 15198469

[34] Caldwell TM, Jorm AF Mental health nurses' beliefs about likely outcomes for people with schizophrenia or depression: a comparison with the public and other healthcare professionals. The Australian and New Zealand Journal of Mental Health Nursing. 2001;10(1):42-54 10.1046/j.1440-0979.2001.00190.x 11421972

[35] Hugo M Mental health professionals' attitudes towards people who have experienced a mental health disorder. Journal of Psychiatric and Mental Health Nursing. 2001;8(5):419-25 10.1046/j.1351-0126.2001.00430.x 11882162

[36] Ross CA, Goldner EM Stigma, negative attitudes and discrimination towards mental illness within the nursing profession: a review of the literature. Journal of Psychiatric and Mental Health Nursing. 2009;16(6):558-67 10.1111/j.1365-2850.2009.01399.x 19594679

[37] Lauber C, Nordt C, Braunschweig C, Rossler W Do mental health professionals stigmatize their patients? Acta Psychiatrica Scandinavica Supplementum. 2006;(429):51-9 10.1111/j.1600-0447.2005.00718.x 16445483

[38] Stull LG, McGrew JH, Salyers MP, Ashburn-Nardo L Implicit and explicit stigma of mental illness: attitudes in an evidence-based practice. The Journal of Nervous and Mental Disease. 2013;201(12):1072-9 10.1097/NMD.0000000000000056 24284643 PMC4031039

[39] Björkman T, Angelman T, Jönsson M Attitudes towards people with mental illness: a cross-sectional study among nursing staff in psychiatric and somatic care. Scandinavian Journal of Caring Sciences. 2008;22(2):170-7 10.1111/j.1471-6712.2007.00509.x 18489686

[40] Munro S, Baker JA Surveying the attitudes of acute mental health nurses. Journal of Psychiatric and Mental Health Nursing. 2007;14:196–202. 10.1111/j.1365-2850.2007.01063.x 17352783

[41] Tsai J, Salyers MP, Lobb AL Recovery-oriented training and staff attitudes over time in two state hospitals. The Psychiatric Quarterly. 2010;81(4):335-47 10.1007/s11126-010-9142-2 20589429

[42] Magliano L, Read J, Sagliocchi A, Patalano M, D'Ambrosio A, Oliviero N Differences in views of schizophrenia during medical education: a comparative study of 1st versus 5th-6th year Italian medical students. Social Psychiatry and Psychiatric Epidemiology. 2013;48(10):1647-55 10.1007/s00127-012-0610-x 23117816

[43] Naeem F, Ayub M, Javed Z, Irfan M, Haral F, Kingdon D Stigma and psychiatric illness. A survey of attitude of medical students and doctors in Lahore, Pakistan. Abbottabad, Pakistan: Journal of Ayub Medical College (JAMC) 2006;18(3):46-9 17348313

[44] Borkin JR, Steffen JJ, Ensfield LB, Krzton K, Wishnick H, Wilder K, et al Recovery Attitudes Questionnaire: Development and evaluation. Psychiatric Rehabilitation Journal. 2000;24(2):95-102 10.1037/h0095112

[45] Diamond RJ Recovery from a psychiatrist's viewpoint. Postgraduate Medicine. 2006; Spec No:54-62 17128661

[46] Harding CM, Zahniser JH Empirical correction of seven myths about schizophrenia with implications for treatment. Acta Psychiatrica Scandinavica. 1994;90:140-6. 10.1111/j.1600-0447.1994.tb05903.x 7879636

[47] Bonin JP, Lesage A, Ricard N, Demers M, Morissette R, Benoit D Empowering the staff in long-stay wards. Can J Psychiatry. 1998 12 43(10):1054-5 9868578

[48] Kingdon D, Sharma T, Hart D What attitudes do psychiatrists hold towards people with mental illness? Psychiatric Bulletin. 2004;28: 401–406. 10.1192/pb.28.11.401

[49] Thornicroft G, Tansella M The mental health matrix: a manual to improve services. Cambridge: Cambridge University Press, 1999 10.1017/CBO9780511549557

[50] Gordon SE, Huthwaite MA, Short JA, Ellis PM Discovering stigma through recovery teaching. Australas Psychiatry. 2014;22(2):186-9 10.1177/1039856213519145 24425799

[51] Rudnick A, Eastwood D Psychiatric rehabilitation education for physicians. Psychiatric Rehabilitation Journal. 2013;36(2):126-7 10.1037/h0094985 23750768

[52] Collège Royal des Médecins et Chirurgiens du Canada. Exigences de la formation spécialisée en psychiatrie - Version 2.0. 2016 http://www.royalcollege.ca/rcsite/documents/ibd/psychiatry-str-f [Accessed October 18, 2018].

[53] Gambino M, Pavlo A, Ross DA Recovery in Mind: Perspectives from Postgraduate Psychiatric Trainees. Academic Psychiatry: the Journal of the American Association of Directors of Psychiatric Residency Training and the Association for Academic Psychiatry. 2016;40(3):481-8. 10.1007/s40596-015-0414-x 26791016

[54] Papish A, Kassam A, Modgill G, Vaz G, Zanussi L, Patten S Reducing the stigma of mental illness in undergraduate medical education: a randomized controlled trial. BMC Medical Education. 2013;13:141 10.1186/1472-6920-13-141 24156397 PMC3828029

[55] Randall M, Romero-Gonzalez M, Gonzalez G, Klee A, Kirwin P Competency of psychiatric residents in the treatment of people with severe mental illness before and after a community psychiatry rotation. Academic Psychiatry: the Journal of the American Association of Directors of Psychiatric Residency Training and the Association for Academic Psychiatry. 2011;35(1):15-20. 10.1176/appi.ap.35.1.15 21209402

[56] Battersby L, Morrow M Challenges in implementing recovery-based mental health care practices in psychiatric tertiary care. Canadian Journal of Community Mental Health. 2012;31(2):103-17 10.7870/cjcmh-2012-0016

[57] Centre d’études sur la réadaptation, le rétablissement et l’insertion sociale (CÉRRIS). 2018 https://criusmm.ciusss-estmtl.gouv.qc.ca/fr/recherche/centres-detudes/centre-detudes-sur-la-readaptation-le-retablissement-et-linsertion-sociale-cerris-0 [Access date on May 19, 2020]

[58] Chen SP, Krupa T, Lysaght R, McCay E, Piat M Development of a recovery education program for inpatient mental health providers. Psychiatric Rehabilitation Journal. 2014;37(4):329-32. 10.1037/prj0000082 24978621

[59] Bélanger S, Briand C, Provencher H, Gilbert M La mise en place et le maintien de pratiques axées sur le rétablissement : guide d’accompagnement de la direction de la santé mentale du Québec. Ministère de la Santé et des Services Sociaux. 2017 Available at : https://publications.msss.gouv.qc.ca/msss/fichiers/2017/17-914-03W.pdf [Accessed November 20, 2018].

[60] Piat M, Sofouli E, Sabetti J, et al Protocol for a mixed studies systematic review on the implementation of the recovery approach in adult mental health services. BMJ Open. 2017;7(8). 10.1136/bmjopen-2017-017080 PMC5724147 28855202

[61] Mental Health Commission of Canada. Opening minds Available at: https://www.mentalhealthcommission.ca/English/opening-minds [20/11/2018].

[62] Burgess P, Pirkis J, Coombs T, Rosen A Assessing the value of existing recovery measures for routine use in Australian mental health services. The Australian and New Zealand Journal of Psychiatry. 2011;45(4):267-80. 10.3109/00048674.2010.549996 21314238

[63] Chen SP, Krupa T, Lysaght R, McCay E, Piat M The development of recovery competencies for in-patient mental health providers working with people with serious mental illness. Administration and Policy in Mental Health. 2013;40(2):96-116. 10.1007/s10488-011-0380-x 22009447

[64] Modgill G, Patten SB, Knaak S, Kassam A, Szeto ACH Opening Minds Stigma Scale for Health Care Providers (OMS-HC): Examination of psychometric properties and responsiveness. BMC Psychiatry. 2014;14(1):120 10.1186/1471-244X-14-120 24758158 PMC4024210

